# Pharmacophore modeling for identification of anti-IGF-1R drugs and *in-vitro* validation of fulvestrant as a potential inhibitor

**DOI:** 10.1371/journal.pone.0196312

**Published:** 2018-05-22

**Authors:** Samra Khalid, Rumeza Hanif, Ishrat Jabeen, Qaisar Mansoor, Muhammad Ismail

**Affiliations:** 1 Atta-ur-Rahman School of Applied Biosciences, National University of Sciences and Technology, Islamabad, Pakistan; 2 Northern Institute for Cancer Research, Newcastle upon Tyne Hospitals NHS Foundation Trust, The Medical School, University of Newcastle upon Tyne, Newcastle upon Tyne, United Kingdom; 3 Research Center for Modeling & Simulation (RCMS), National University of Sciences and Technology, Islamabad, Pakistan; 4 Institute of Biomedical and Genetic Engineering (IBGE), KRL Hospital, Islamabad, Pakistan; University of South Alabama Mitchell Cancer Institute, UNITED STATES

## Abstract

Insulin-like growth factor 1 receptor (IGF-1R) is an important therapeutic target for breast cancer treatment. The alteration in the IGF-1R associated signaling network due to various genetic and environmental factors leads the system towards metastasis. The pharmacophore modeling and logical approaches have been applied to analyze the behaviour of complex regulatory network involved in breast cancer. A total of 23 inhibitors were selected to generate ligand based pharmacophore using the tool, Molecular Operating Environment (MOE). The best model consisted of three pharmacophore features: aromatic hydrophobic (HyD/Aro), hydrophobic (HyD) and hydrogen bond acceptor (HBA). This model was validated against World drug bank (WDB) database screening to identify 189 hits with the required pharmacophore features and was further screened by using Lipinski positive compounds. Finally, the most effective drug, fulvestrant, was selected. Fulvestrant is a selective estrogen receptor down regulator (SERD). This inhibitor was further studied by using both *in-silico* and *in-vitro* approaches that showed the targeted effect of fulvestrant in ER+ MCF-7 cells. Results suggested that fulvestrant has selective cytotoxic effect and a dose dependent response on IRS-1, IGF-1R, PDZK1 and ER-α in MCF-7 cells. PDZK1 can be an important inhibitory target using fulvestrant because it directly regulates IGF-1R.

## Introduction

Insulin-like growth factor type-1 receptor (IGF-1R), a trans-membrane tyrosine kinase, is involved in normal body growth and development [1]. It has two extracellular ligand binding domains, alpha (α) and beta (β) [2, 3]. IGF-1R is regulated by the binding of ligands, insulin-like growth factors such as IGF-1, to process cell proliferation and differentiation [4–6]. Previous *in vivo* and *in vitro* studies have linked higher levels of IGF-1R and its ligands with various types of cancer development and progression including breast cancer [7–10], prostate cancer [11], myeloma [12] and colon cancer [13, 14]. About 50% of the breast tumors have been reported with an over expression of IGF-1R [15]. Although several clinical trials inhibiting this receptor have been completed but unfortunately monoclonal antibodies and tyrosine kinase inhibitors targeting IGF-1R failed in phase III clinical trials for several reasons [16–18]. The activation of IGF-1R upon ligand binding induces phosphorylation of an adopter protein insulin receptor substrate-1 (IRS-1) which is also linked to various cancer subtypes [6, 19]. The signaling cascade of IGF-1R begins by the activation of several downstream mediators such as phosphoinositide3 kinase-serine/threonine protein kinases (PI3k-Akt), mitogen activated kinase-extracellular signal regulated kinase (MEK-ERK) and ataxia telangiectasia mutated-ataxia telangiectasia Rad3 related (ATM-ATR) pathways [19–23]. Deregulation of these pathways induce over-expression of estrogen receptor-alpha (ER-α) which indirectly stimulates the activation of PDZ domain containing 1 (PDZK1) gene expression [24].

PDZK1 protein, also known as NHERF (Na^+^/H^+^ exchange regulatory factor), interacts with phospholipase C-β (PLC-β) and contributes to the regulation of G-protein coupled receptor (GPCR)-mediated signaling [25]. The increased expression of PDZK1 leads to the subsequent phosphorylation of ERK1/2 and calcium ions (Ca^2+^) signaling in response to somatostatin (SST) and IGF-1R [25, 26]. The direct molecular interaction between IGF-1R and PDZK1 enhances expression of ER-α associated with breast cancer metastasis [26]. The IGF-1R pathway facilitates loss of function mutations of multiple tumor suppressor and oncogenes including breast cancer susceptibility genes 1/2 (BRCA1/2), p53 and mouse double minute 2 homolog (Mdm2) which drastically influence resistance to apoptosis [20, 27]. This study focused on the identification of inhibitors against IGF-1R by using well-known *in-silico* approaches, i.e. pharmacophore modeling [28], virtual screening (VS) [29] and continuous hybrid Petri net (PN) [30].

Ligand based pharmacophore modeling is used to generate a set of chemical compounds with required pharmacophore features such as hydrophobic (HyD), aromatic (Aro), hydrogen bond acceptors (HBAs) or donors (HBDs), cations, and anions [31–33]. This ligand based modeling defines the supramolecular interactions of the above mentioned features with the desired molecular target to block its biological activity [32]. In order to identify the potential inhibitory drugs that can bind to the target, virtual screening (VS) is performed. VS is a computational drug discovery technique used to screen these chemical structures which are most likely to bind to one or more active ligands [33, 34]. This study was further enriched with continuous PN modeling [35], which allows us to analyze the delay parameters of the involved entities (proteins/genes). PN is a graph theoretical approach, which has been successfully implemented for the models and analysis of homeostatic/pathological response of IGF-1R associated network with breast cancer.

The computational modeling provides a new insight to analyze the complex dynamical interactions among genes and proteins related to multifactorial diseases such as cancer. We have deployed a molecular drug screening approach which screened the drugs that bind to the active site of target molecules and inhibit their activity. The purpose of this study is to identify new IGF-1R inhibitors by using bioinformatics tools for breast cancer treatment. One of the inhibitors, fulvestrant, was validated by *in-vitro* experiments to understand the changes in expression levels of genes and proteins are involved in the breast cancer signaling pathway.

## Experimental methods

Bioinformatics deals with computational approaches which have been used to identify the complex biological functions and drug designing. Such *in-silico* approaches are used to design the drug targets and solve various biomedical problems [36]. Furthermore, the activity of potent inhibitor is further analyzed by *in-vitro* experiments to validate the effect of such inhibitors on genes/proteins involved in signaling pathway related to breast cancer. The methodology of both *in-silico* and *in-vitro* approaches of the current study are explained below.

### Dataset collection

Pharmacophore modeling was first introduced by Paul Ehrlich to demonstrate the effectiveness of ligand-protein interaction by providing chemical features of the compounds [28]. The pharmacophore model generation demands for an accurate and precise input data [28]. In dataset collection, the following criteria was considered: (i) To characterize the activity of binding interaction of compounds with receptor; (ii) inhibitory potency (IC_50_) in a micromolar (μM) range; (iii) the most, least and inactive compounds must be included in the training set.

**Fig 1:**
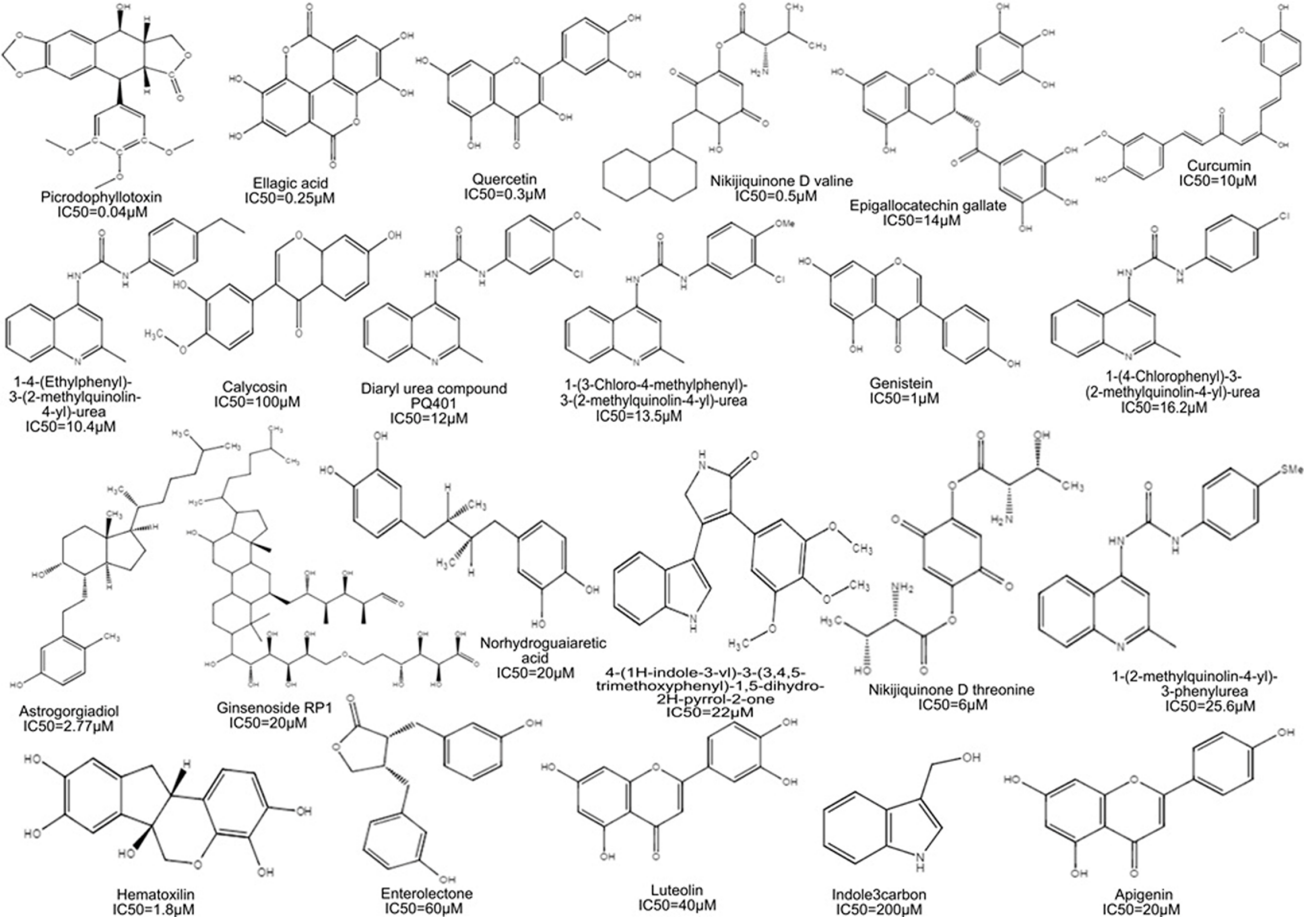
Dataset: Chemical structures and activities of IGF-1R inhibitors in the training set. The dataset consists of chemical structures and activities of IGF-1R inhibitors of four most active (IC_50_ ≤ 0.5μM), thirteen moderately active (IC_50_ ≤ 20μM), four less active (IC_50_ ≤ 60μM) and two were considered as inactive (IC_50_ > 60μM) compounds.

According to the above mentioned criteria, a series of active chemical compounds were retrieved from the literature along with their inhibitory potencies (IC_50_) range from 0.04μM to 200μM [37–52]. The IC_50_ values of all the desired compounds were measured using cell viability and tyrosine kinase biological assays [37–52]. The 2-Dimensional (2D) structure of the compounds was drawn using ChemDraw program (version 8.0) [53] as shown in Fig 1. The collected dataset was classified on the basis of IC_50_ value, that is, most active (IC_50_ ≤ 0.5μM), moderately active (IC_50_ ≤ 20μM), less active (IC_50_ ≤ 60μM) and inactive (IC_50_ > 60μM).

### Conformational dataset generation

The conformations of the dataset were generated by using default settings in software MOE version 2007.09 [54]. MOE is comprehensive drug discovery software used ligand and structure-based pharmacophore modeling. However, in this study we used ligand-based pharmacophore modeling to reveal the chemical features important for the compounds activity against IGF-1R. The Scientific Vector Language (SVL) command-line is provided by MOE that integrates with database browsers which helps in the formation of packed conformation dataset [55]. The conformational search module in MOE was used to generate minimum energy conformations of each active and inactive compound.

### Ligand-based pharmacophore modeling

**Fig 2:**
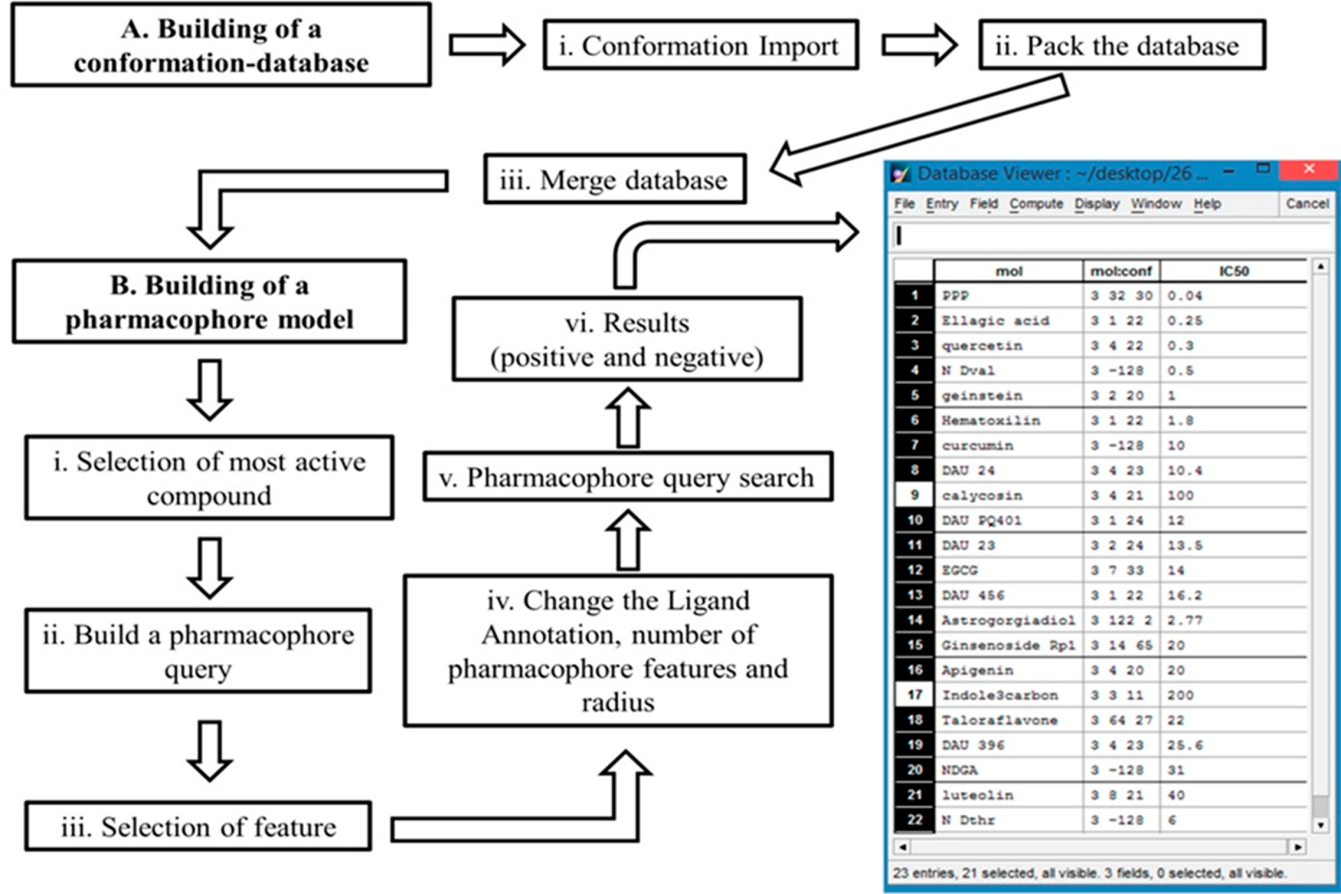
Workflow diagram presenting the ligand based pharmacophore modeling. **(A)** In the first part of pharmacophore modeling is the building of conformational-database of 23 chemical compounds in MOE (i) to compute confirmations by confirmation import. (ii) Pack the conformational data-base in the MOE commandline. (iii) After packing merge the conformational data-base with original dataset of 23 compounds. **(B)** In the second part of building of pharmacophore modeling (i) choose an active compound such as picropodophyllin (ii) to build a pharmacophore query. (iii) Open the pharmacophore query editor to select the feature by (iv) changing the ligand annotation, number and radius of pharmacophore features under query rendering to build the pharmacophore model. (v) A click on search starts the query search to confirm the (vi) results of 21 active and 2 inactive compounds.

A conformational training dataset of 23 chemical compounds was implemented in MOE to generate pharmacophore hypothesis. The methodology for ligand based pharmacophore modeling is explained in Fig 2. The dataset consisted of 21 active and 2 inactive compounds were employed to influence the quality of pharmacophore model depending on the two basic values are specificity and sensitivity. Active compounds have well known pharmacokinetic properties such as drug absorption, distribution, metabolism and excretion that directly bind to targeted receptors while inactive compounds had poor binding affinity [55]. In the present study, only active compounds were selected with common features such as HBDs, HBAs, Aro, HyD, number of non-polar atoms and rotatable bonds. The developed pharmacophore model was selected based on molecular sequence and low RMSD (see supporting Information S1 Table).

### Virtual screening (VS)

The pharmacophore model developed using the most active compound picropodophyllin (0.04 μM) was further used for screening the large library of World Drug Bank (WDB) database [29]. After VS, 2534 compounds were first screened by Lipinski’ positive compounds < 5 HBD groups, < 10 HBA groups and Lipinski drug-likeness [56]. Finally, drug-like compounds with the features such as total number of rotatable bonds, number of rings and topological polar surface area [57–59] were further screened to examine final hit compounds.

### Petri net (PN) model generation

The PN model was generated using the SNOOPY tool (version 2.0) [60] to model continuous dynamics based on trajectories involved in the signaling pathway related to breast cancer. In this study, we have implemented a graph theoretical hybrid PN approach which uses the kinetic logic formalism based on signaling proteins (represented as places) and interactions among proteins/genes (represented as transitions). These dynamics are specified as mass action and Michaelis-Menten equations [60]. The value of kinetic parameter is developed by the firing of transition with infinite number of tokens from source place on a target place. The firing rate is enabled to produce the expression dynamics with the same initial marking executed by multiple time simulations. The marking of places within tokens to describe the concentration of proteins are modeled to monitor the dynamics of parameters in a signaling devised by [61]. We analyzed the PN model with 10, 50 and 100 time units in simulation runs. The data was obtained by high throughput technologies (western blots, microarrays, immunohistochemistry) of several studies [6, 21, 62–66] used to validate the expression levels of individual entity involved in ER-α associated breast cancer progression.

### *In-vitro* cell culture

MCF-7 cells were maintained in Dulbecco’s Modified Eagle's medium (DMEM) (Invitrogen, Paisley, UK) supplemented with 10% (v/v) foetal calf serum (FCS). Cells were incubated at 37°C with 5% CO_2_ in a humidified incubator. Before treatment with fulvestrant the medium was changed and cells were cultured after 3–5 days.

### Cell viability assay

1×10^5^ MCF-7 cells, obtained from the American Type Culture Collection (ATCC, Manassas, VA), were platted in a 96-well plate for 24 hours (hrs). The cells were washed with phosphate buffer saline (PBS) and treated with different concentrations (0.001–1 μM mL^−1^) of fulvestrant (Faslodex, AstraZeneca, USA) for 48 hrs. Later, 250μg mL−1 MTT (Sigma-Aldrich, Biotechnology, USA) was added to each well and incubated for 4 hrs at 37°C. The cultured media was replaced with 100μL of Dimethyl sulfoxide (DMSO) (Sigma-Aldrich, Biotechnology, USA) after incubation. The absorbance was recorded at a wavelength of 570nm in a microplate reader (Techno Service, AMP Platos R496, Egypt). The %age viability of cells were calculated by using the following equation: Cell viability rate (%) = [1- (absorbance of test samples/absorbance of the control group)] x 100%. The 50% inhibitory concentration (IC_50_ values) was calculated from the plotted absorbance data for the dose–response curve.

### Reverse transcription and quantitative real-time PCR (q-RT-PCR)

RNA was isolated from treated MCF-7 cells by Trizol reagent (Invitrogen, Waltham, USA). From 1 μg purified RNA, cDNA was synthesized using RevertAid™ Reverse Transcriptase (Thermo Scientific) and oligodT (Fermentas, Waltham, USA) in cDNA kit (Invitrogen, Los Angeles, USA) according to manufacturerʹs instructions. For real time PCR detection of IGF-1R [67], PDZK1 [68] and ER-α [69] (primer sequences shown in Table 1), 1 μg of cDNA was amplified using a SYBR Green PCR kit (Invitrogen, Los Angeles, USA) with 0.3μM of forward and reverse primers. The PCR program was run on real time PCR (Sansure Biotech Inc, Changsha, China) and conditions were started with initial denaturation at 95°C for 10 min followed by 40 PCR cycles (95°C for 15 seconds, 60°C for 20 seconds and 72°C for 40 seconds) and final extension at 72°C for 5 minutes. In all q-RT-PCR experiments, β-actin [70] was amplified as a housekeeping gene to the relative mRNA of target genes IRS-1, IGF-1R, PDZK1 and ER-α to calculate ΔΔCt values.

**Table 1:** List of primer sequences used for q-RT-PCR amplification.

S.No.	Genes	Sequences (5’-3’)	Base pair
1	IGF-1R (F)	GGGAATGGAGTGCTGTATG	19
	IGF-1R (R)	CACAGAAGCTTCGTTGAGAA	20
2	PDZK1 (F)	CCCACAGTACAGCCTCACATT	21
	PDZK1 (R)	CACATGGTGAATGGTTTCCA	20
3	ER-α (F)	CCACCAACCAGTGCACCATT	20
	ER-α (R)	GGTCTTTTCGTATCCCACCTTTC	23
4	β-actin (F)	ACCTTCAACACCCCAGCCATGTACG	25
	β-actin (R)	CTGATCCACATCTGCTGGAAGGTGG	25

### Western blotting

MCF-7 cells were treated with fulvestrant (0.001–0.01 μM) for 48hrs. Cells were washed with PBS and collected to prepare cell lysate with radioimmunoprecipitate assay (RIPA) buffer: 50mM Tris-hydrochloride (HCl) pH 7.5, 150mM sodium chloride (NaCl), 1mM Ethylenediaminetetraacetic acid (EDTA), 1% Nonyl phenoxypolyethoxylethanol (NP-40) (v/v), 0.25% sodium deoxycholate (C_24_H_40_O_4_) (w/v), 1μgml-1 pepstatin (C_34_H_63_N_5_O_9_), 1μgml-1 aprotinin, 1μgml-1 leupeptin (C_20_H_38_N_6_O_4_), 2mM sodium orthovanadate (Na_3_VO_4_), 2mM sodium fluoride (NaF) and 2 mM PMSF (Phenylmethylsulphonyl fluoride) (Sigma-Aldrich, Dorset, United Kingdom) [71]. The protein was quantified by bicinchonic acid (BCA) assay as a standard (Thermo Scientific, Lough borough, UK) [72]. Proteins in the cell lysates and media were separated by 12% SDS-PAGE (sodium dodecyl sulfate-polyacrylamide gel electrophoresis). Proteins were transferred to 0.45μm nitrocellulose membrane (Schleicher & Schuell, Krackeler, USA) and probed with antibodies: esrogen receptor-alpha (ER-α) 1:2000 (sc-8005) (Santa Cruz, Biotechnology, California); anti-PDZK1 1:3000 (#HPA006155); IRS-1 1:2000 (#3407); type I IGF receptor (#3027) (Cell Signaling Technologies, Hitchin, United Kingdom) and GAPDH (sc-25778) (Santa Cruz Biotechnology, Heidelberg, Germany). Membranes were blocked with 5% milk-TBST (25 mM Tris pH7.4, 150 mM NaCl, 0.1% Tween 20) and incubated with 1:5000 secondary antibodies (Cell Signaling Technologies, Hitchin, United Kingdom). Proteins were visualized by SuperSignal West Dura Substrate (Thermo Scientific, Rockford, USA) and exposed to X-ray film.

### Statistics

The OD of X-ray film protein bands was measured by densitometric quantification with LabWorks 4.0 software, adjusted and normalized to GAPDH or total corresponding proteins (UVP, Inc, Cambridge, United Kingdom). Data was expressed as %age for the maximum amount of molecule detected in each experiment. Statistical differences between groups were performed by one-way analysis of variance (ANOVA) or un-paired ‘t’ test using the GraphPad PRISM statistic software (version 7.0) (GraphPad Software, La Jolla, California USA). Statistically significant P-values (< 0.05) were considered.

## Results

### Pharmacophore model generation and evaluation

**Fig 3:**
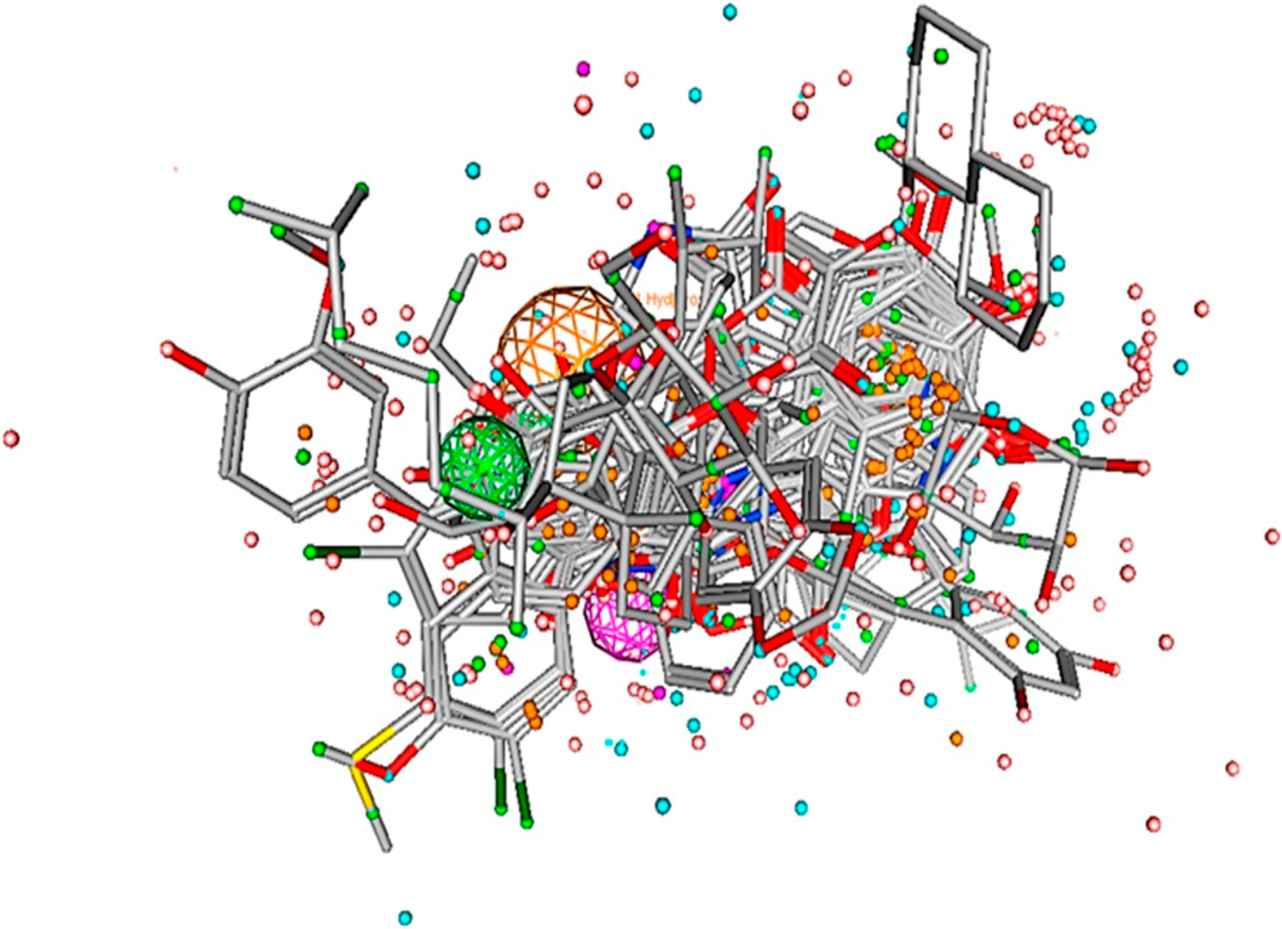
Pharmacophore model and aligned ligands. The pharmacophore model of IGF-1R inhibitors are generated by MOE module with set of aligned active compounds. Pharmacophore model was generated with three chemical features such as aromatic hydrophobic (HyD/Aro); hydrophobic (HyD) and hydrogen bond acceptor (HBA) represented by orange, green and purple, respectively.

**Fig 4:**
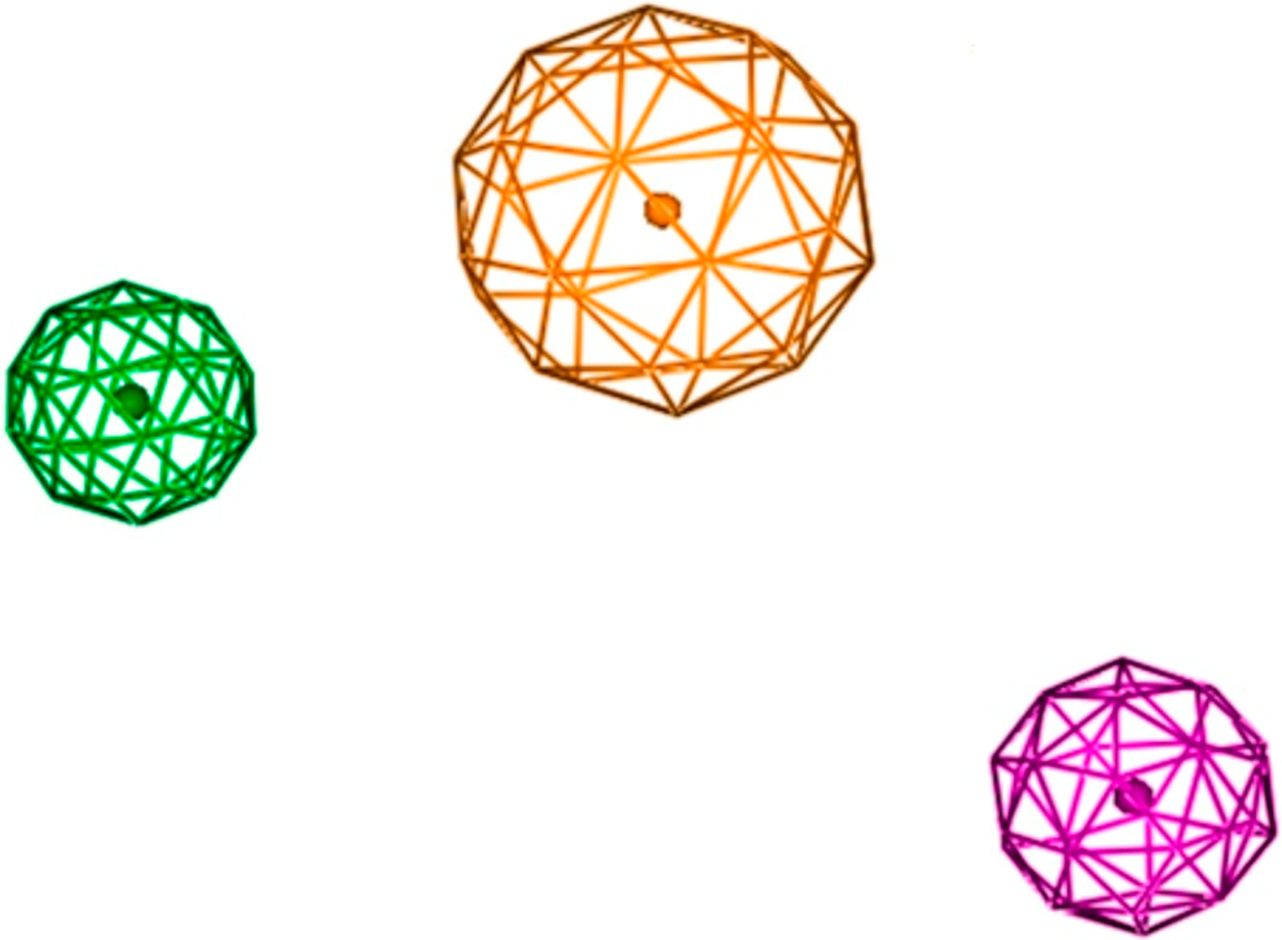
Pharmacophore features. Pharmacophore features are defined as orange, green and purple colored represented by F1-aromatic hydrophobic (HyD/Aro), F2-hydrophobic (HyD) and F3-hydrogen bond acceptor (HBA).

Pharmacophore model was generated from the dataset of 23 compounds with inhibitory potency ranging between 0.04 to 200μM (Fig 3). These compounds have the potential to inhibit IGF-1R which interacts with various transcription factors including ER-α, ER-β, Vascular Endothelial Growth Factor Receptor-3 (VEGFR‐3), human epidermal growth factor receptor 2 (HER2), IRS-1, Mitogen-activated protein kinase (MAPK) and PI3K/Akt [37–52]. Several studies have elucidated over-expression of IGF-1R in breast cancer progression [4, 6, 20, 21, 73, 74]. The statistical parameters, root mean square deviation (RMSD) (0.01–0.89) (shown in S1 Table as supporting information) of 21 active compounds were generated to build the pharmacophore model (shown in Fig 3) using the tool, molecular operating environment (MOE) version 2007.09 [54]. The parametric values included three biological features along with radius (R): F1; HyD/Aro (1.4), F2; HyD (0.8) and F3; HBA (0.9) (shown in Fig 3) represented by orange, green and purple coloured circles, respectively (shown in Fig 4). The dataset is divided into three categories: the most active (IC_50_ ≤ 0.5μM), moderately active (IC_50_ ≤ 20μM) and less active (IC_50_ ≤ 60μM). The inactive compounds had poor pharmacokinetic properties such as drug absorption, distribution, metabolism, excretion and drug-drug interaction which were undetected to prove the effectiveness of our model.

The selectivity of our model has been evaluated by the set of both active (truely identified as positive) and inactive (truely identified as negative) compounds. The active compounds with diverse scaffolds were matched to features of pharmacophore model while the inactive compounds were not retrieved by the model. The enrichment of pharmacophore model has been assessed by two basic values, selectivity and specificity [75]. The model with accuracy rate of 1, showed the best predictive ability with high selectivity and specificity which are defined by the retrieval of active and inactive compounds, respectively. The distances between pharmacophore features have been identified as: (F1-HyD/Aro-F2- HyD: 3.75Å), (F1-HyD/Aro-F3-HBA: 4.99Å) and (F2-HyD-F3-HBA: 6.79Å), shown in Table 2. The distances shown here represent how pharmacophore features are participating to interact with different chemical groups to identify potential ligands against the target IGF-1R.

**Table 2:** Pharmacophore features with distance constraints (Å). Pharmacophore features have mutual distances between aromatic hydrophobic (HyD/Aro), hydrophobic (HyD) and hydrogen bond acceptor (HBA).

**Feature types**	**HyD/Aro**	**HyD**	**HBA**
**HyD/Aro**	0 Å	3.75 Å	4.99 Å
**HyD**	3.75 Å	0 Å	6.79 Å
**HBA**	4.99 Å	6.79 Å	0 Å

### Database screening

**Fig 5:**
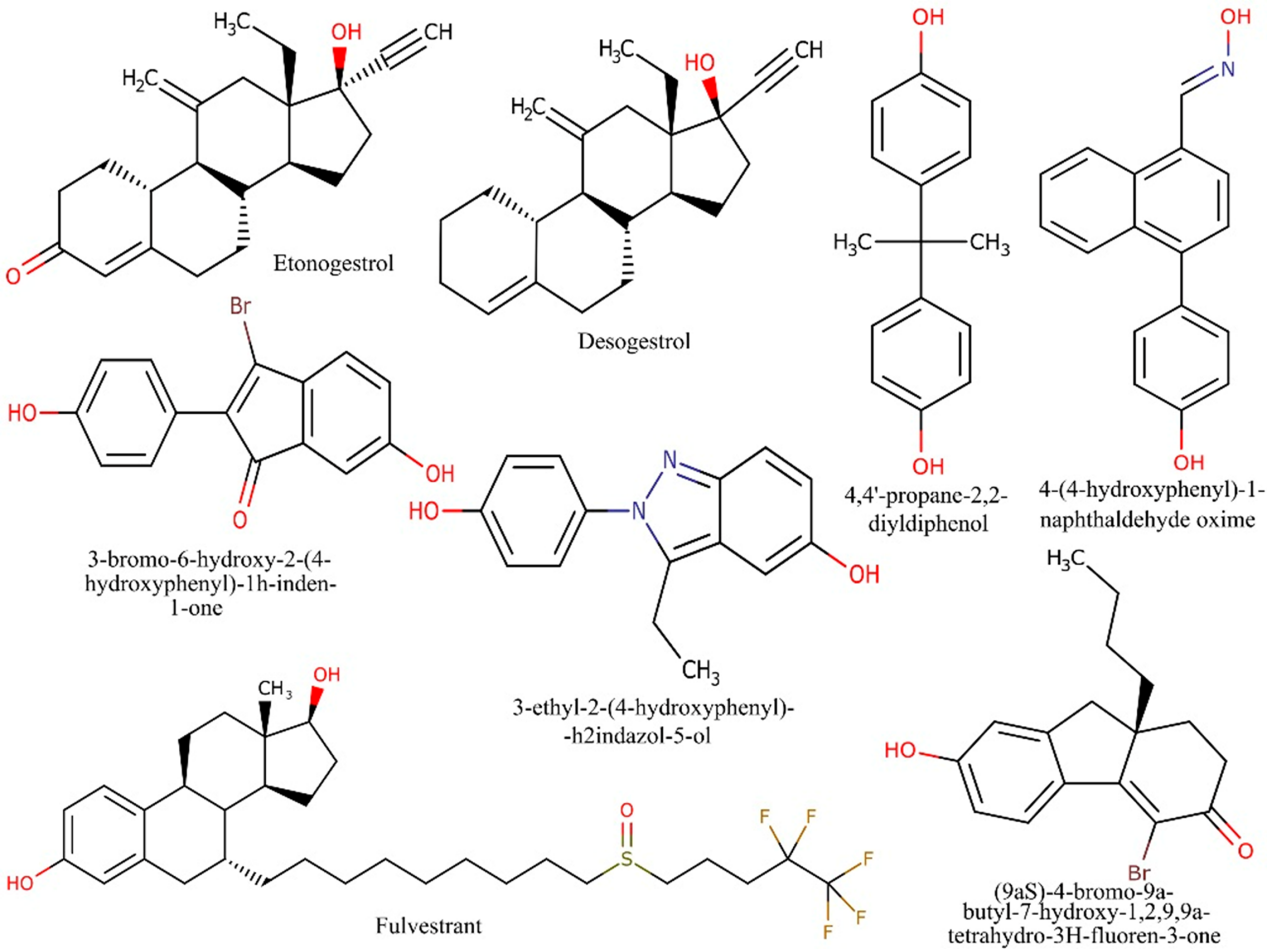
Potential hits against IGF-1R. Out of Potential 8 hits, fulvestrant is selected for biological evaluation.

Virtual screening (VS) was perform using the developed pharmacophore model against world drug bank (WDB) database [76] to identify final eight hit compounds as shown in Fig 5. Some of these compounds such as etonogestrol (DB00294), desogestrol (DB00304) and fulvestrant (DB00947) have anti-estrogenic activity while others belong to the class of organic compounds: (9aS)-4-bromo-9a-butyl-7-hydroxy-1,2,9,9a-tetrahydro-3H-fluoren-3-one (DB07757), 4-(4-hydroxyphenyl)-1-naphthaldehyde oxime (DB07150), 3-bromo-6-hydroxy-2-(4-hydroxyphenyl)-1h-inden-1-one (DB07230), 4,4'-propane-2,2-diyldiphenol (DB06973) and 3-ethyl-2-(4-hydroxyphenyl)-2h-indazol-5-ol (DB07712). Etonogestrol and desogestrol are synthetically active metabolites that have high affinity for estrogen receptor (ER) and progesterone receptor (PR) [77, 78]. In the treatment of estrogen receptor positive (ER+) breast cancer, both of these drugs restrain fertility by suppressing the release of luteinizing hormone (LH), one of the contraceptive hormones imperative in ovulation [77, 78]. Fulvestrant has been used as a selective hormone receptor positive (HR+) breast cancer treatment and provides greater control over endocrine therapy resistance [79]. The organic compounds such as DB07757, DB07150, DB07230, DB06973 and DB07712 have transcriptional efficacy against ER-α and ER-β [80–82]. Due to unavailability of these compounds, we finally selected the most active drug fulvestrant in this study which was further evaluated by using both *in-silico* and *in-vitro* experiments. We have also found that fulvestrant is an anti-IGF-1R compound against breast cancer cells and can control tumerogenesis. This identification indicates that *in-silico* based approaches are used to save our time and resources to identify the potential drugs against specific target to control complex diseases such as cancer.

### Analysis of hybrid PN modeling of IGF-1R associated diseased regulatory network

**Fig 6:**
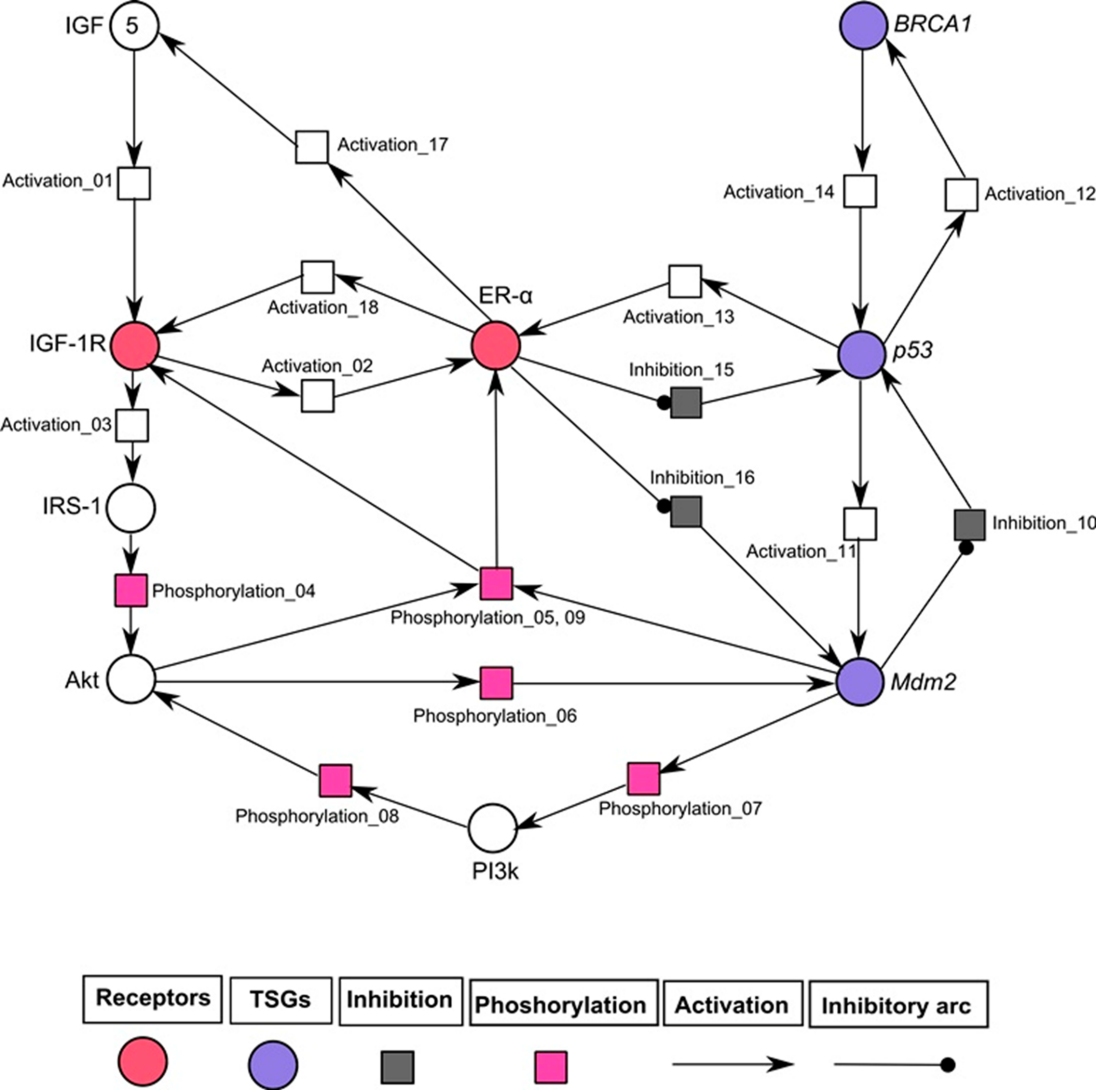
Illustration of diseased IGF-1R associated hybrid PN model. In this PN model, circles (red, blue and white colored) represent continuous places which described the behaviour of entities (IGF, IGF-1R, IRS-1, Akt, PI3k, ER-α and TSGs), while squares (pink, black and white colored) represent the continuous transitions to illustrate the processes of phosphorylation, inhibition and activation. Activation (represented by directed arrows) starts signal from continuous place towards transition while inhibition (represented by inhibitory arcs) stops signal towards continuous transition. The ligand IGF-1 is given with an arbitrary token no. of five. The rate of mass action of continuous transitions of the involved entities is taken as 1.

**Fig 7:**
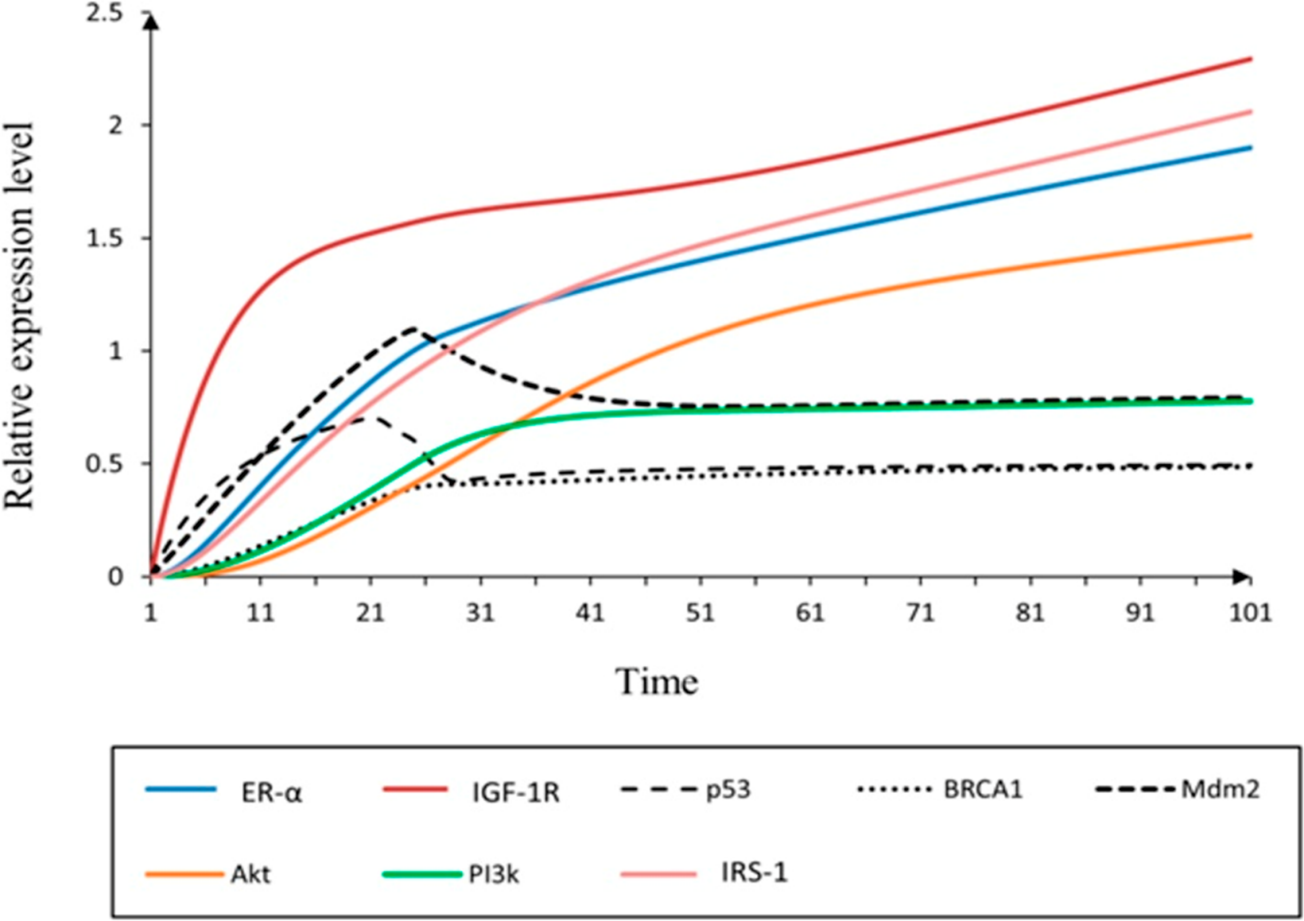
Simulation of un-treated hybrid PN model. The simulation graph represents the relative expression level (*x*-axis) of IGF-1R associated entities with respect to time (*y*-axis). The mutated behaviour of IGF-1R associated breast cancer signaling is observed by the relative low expression levels of *p53*, *BRCA1* and *Mdm2* (dash, round dot and square dot) with the over-expression of IGF-1R (red).

**Fig 8:**
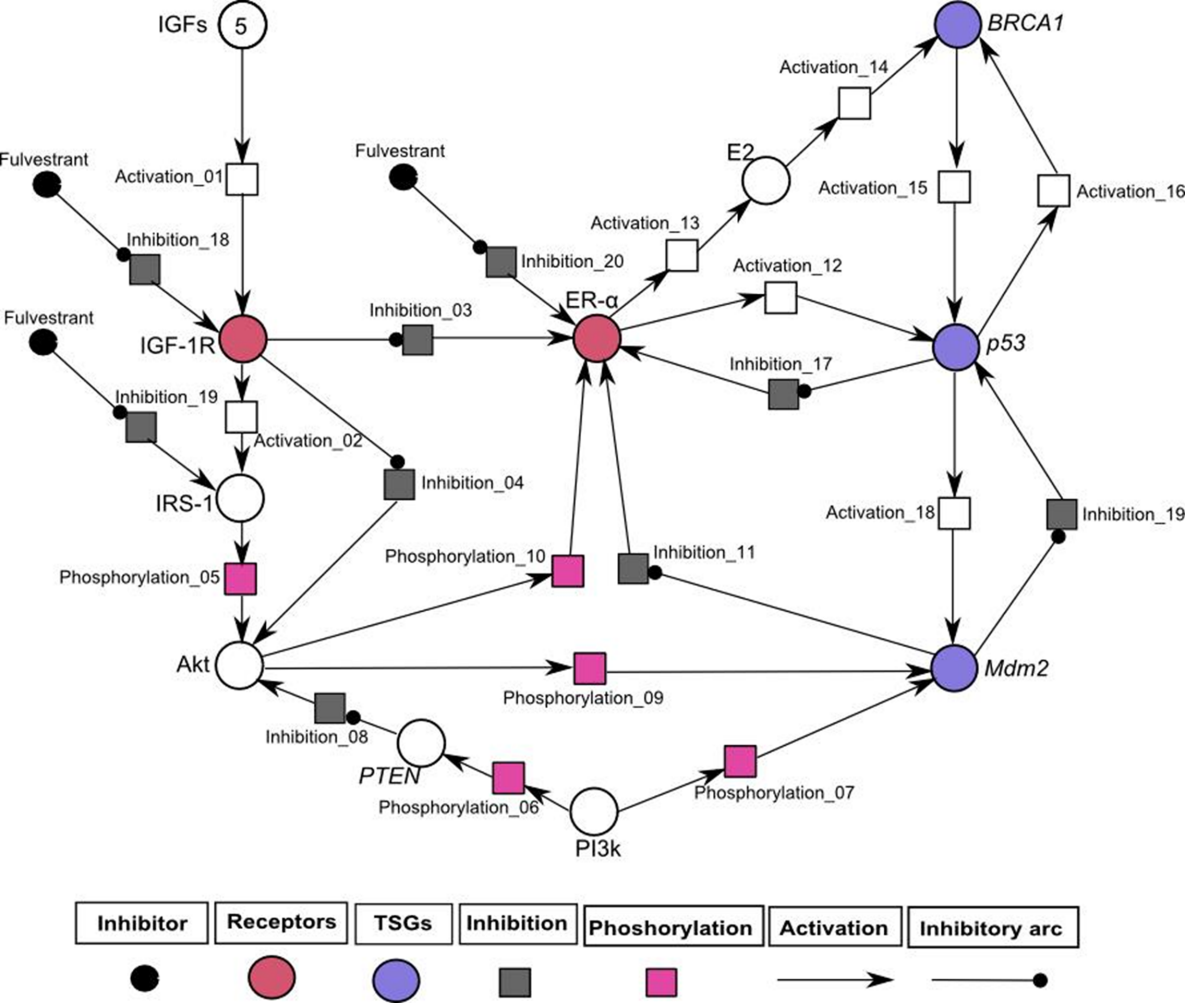
Illustration of IGF-1R associated hybrid PN model demonstrates the inhibitory effect of inhibitor (fulvestrant). In this PN model, circles (red, blue and white colored) represent continuous places which describes the behaviour of entities (IGF, IGF-1R, IRS-1, Akt, PI3k, ER-α and TSGs) and the squares (pink, black and white colored) represent the continuous transitions to illustrate the processes of phosphorylation, inhibition and activation. Activation (represented by directed arrows) starts signal from continuous place towards transition and inhibition (represented by inhibitory arcs) stops signal towards continuous transition. The ligand IGF is given with an arbitrary token number of five. The rate of mass action of continuous transitions of the involved entities is taken as 1.

**Fig 9:**
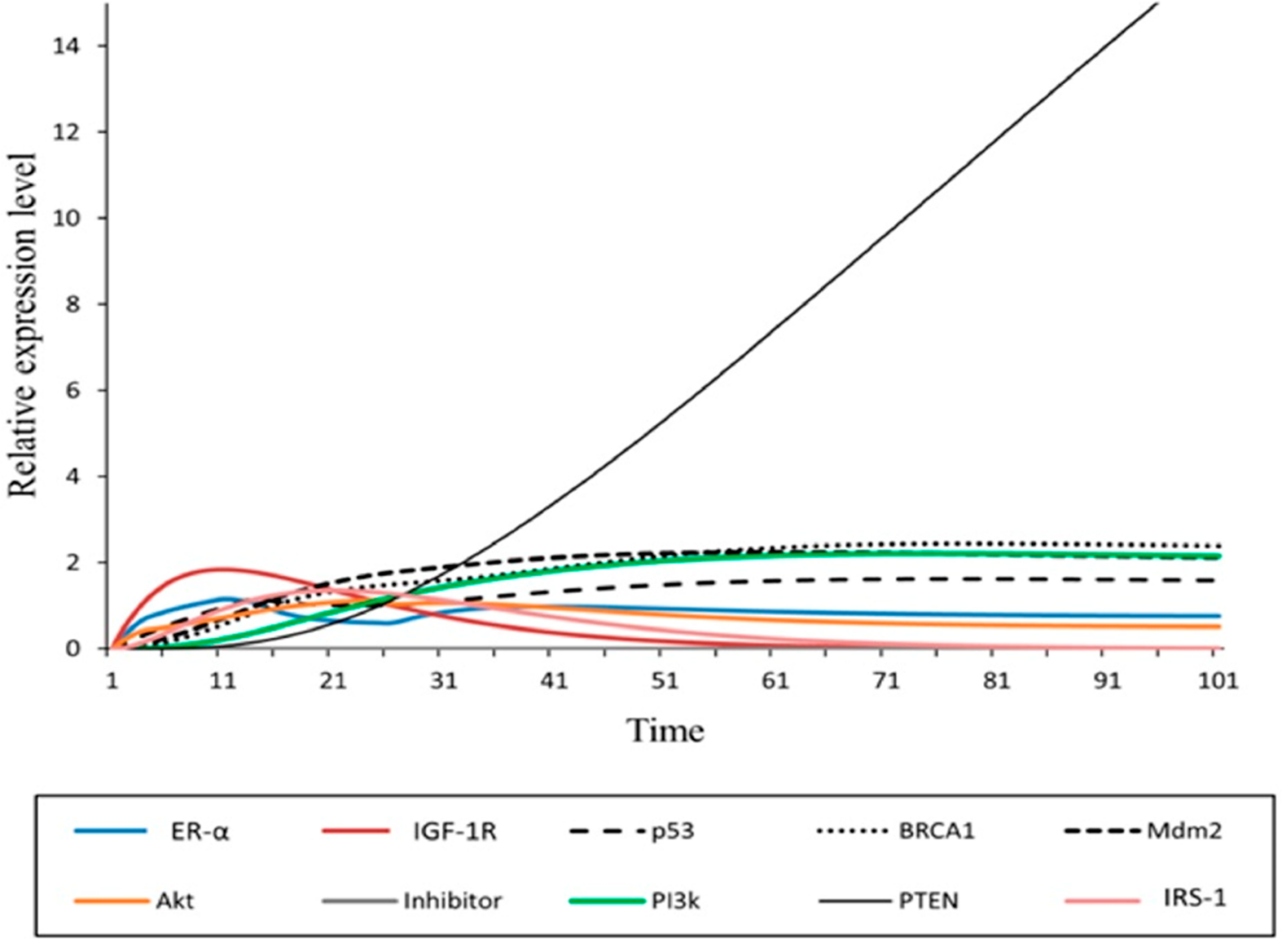
Simulation of treated hybrid PN model. The simulation graph represents the relative expression level (*x*-axis) of IGF-1R associated entities with respect to time (*y*-axis). The effect of inhibitor (fulvestrant) is observed by the relative high expression levels of *p53*, *BRCA1* and *Mdm2* (dash, round dot and square dot) with the down-regulation of IGF-1R (red).

The hybrid PN models were constructed to observe the continuous dynamics of key proteins involved in IGF-1R associated un-treated (diseased) and treated network. Two hybrid PN models and their simulated graphs were observed to represent the mutated (given in Figs 6 and 7) and normal behaviour after treatment (given in Figs 8 and 9). Further the role of entities involved in IGF-1R signaling was evaluated. The analysis of PN model was performed to reveal the time dependent behaviour of each entity involved in diseased regulatory network (Fig 6). The signaling cascade begins by the binding of ligand IGF (token number of 5) with the receptor (IGF-1R) which leads to the phosphorylation of IRS-1. It further initiates the signaling of protein kinase PI3k which is involved in the activation of ER-α through phosphorylation of Akt. Previous studies have demonstrated that the hyper-activity of ER-α is enhanced during the pathogenesis by transcriptional activation of IGF-1 [20, 63, 74, 83]. Fig 6 illustrates that ER-α has positive feedback loop to IGF-1R which is switched on to inhibit the activity of tumor suppressor genes (TSGs) including p53 and Mdm2. *Mdm2* is a key negative regulator of *p53* and regulates the activity of *BRCA1* which maintains the homeostatic function of system. In pathological conditions, loss of function mutations of TSGs can disrupt the function of hormonal and growth factor receptors in signaling network. The continuous parameters of the constructed IGF-1R associated signaling network were selected using tool SNOOPY (version 2.0) [60] by encoding the wet lab observed behaviours in PN modeling. The hybrid PN analysis obtained in this study resulted to identify the increased expression of IGF-1R, IRS-1 and ER-α drastically involved in increased risk of breast cancer metastasis. Our model has predicted that deregulation of IGF-1R associated signaling pathway should be controlled by the inhibition of multiple targets in order to treat breast cancer.

The simulation results of IGF-1R associated diseased hybrid PN model is demonstrated in Fig 7. It shows the continous dynamics of each entity through a simulation graph to observe the relative expression levels with respect to time. It has been observed that increased expression of IGF-1R limits the homeostatic behaviour of TSGs by the inhibitory effect of ER-α [6, 20, 84–86]. The higher levels of IGF-1R and ER-α (represented by red and cyan sigmoid curves) lead to suppression of *p53*, *BRCA1* and *Mdm2* (represented by dash, round dot and square dot curves) which results in the disruption of normal body functions. The IGF-1R is activated (increased level by 2 fold) through the phosphorylated activity of substrate IRS-1 (represented by pink sigmoid curve) to continue processes such as cell proliferation and DNA replication [6, 19]. The feedback regulation of ER-α has achieved by the transcriptional activation/autophosphorylation of kinases including Akt and PI3k (represented by orange and green sigmoid curves). The biological effect of E2-mediated PI3k/Akt activation is up-regulated through ER-α dependent mechanism which stimulates growth in breast cancer cells [87]. So, it is important to note that IGF-1R, IRS-1 and ER-α serve as important inhibitory targets in breast cancer treatment.

### *In-silico* treatment with fulvestrant changed the relative expression levels of proteins involved in IGF-1R associated Hybrid PN modeling

Previously, it has been shown that fulvestrant down-regulates the activity of ER-α and has excellent anti-proliferative efficacy in several cancers dealing with ER+ breast [88], ovarian [89], non-small cell lung [90] and gastric cancer cells [91]. The hybrid PN model was constructed to demonstrate the inhibitory effect of fulvestrant on key entities such as IGF-1R, IRS1 and ER-α involved in IGF-1R associated regulatory network (shown in Fig 8). Fulvestrant has also been to be an anti-IGF-1R drug which further controls the transcriptional activation and auto-phosphorylation of signaling kinases such as Akt (shown in Fig 8). In homeostatic conditions, ER-α and PI3k involved in direct or indirect (through E2) activation of TSGs including p53, BRCA1 and PTEN. *PTEN* act as a negative regulator of Akt that preferentially de-phosphorylate PI3k [92] and results in the regulation of *Mdm2* which induces apoptosis in cancerous cells [93]. The regulatory effect of *Mdm2* has also been achieved by the down-regulating the expression of ER-α which leads the system towards homeostasis.

The treated behaviours of entities were simulated to observe the relative expression levels of IGF-1R associated hybrid PN model (Fig 8) and graph plotted with respect to time is shown in Fig 9). It shows positive network of interaction of *PTEN* (represented by black sigmoid curve) with *p53* which is directly involved in the regulation of cell cycle [94]. The *PTEN*/*p53* complex inhibits PI3k and Akt (represented by green and orange sigmoid curves) signaling pathway that promotes transcriptional activity of *Mdm2* [94, 95]. The inhibitory actions of *p53* (represented by dash sigmoid curve) and *Mdm2* (represented by square dot sigmoid curve) towards ER-α (represented by cyan sigmoid curve) can also be activated by up-regulated expression of *BRCA1* (represented by round dot sigmoid curve). Previous studies have confirmed that regulation of TSGs results in respective activation or deactivation of ER-α [63, 65, 86, 96, 97]. The increased expression of *PTEN* and TSGs has been observed in down-regulating the expression of IGF-1R (represented by red sigmoid curve), IRS-1 (represented by pink sigmoid curve) and ER-α with the help of fulvestrant.

### Comparison analysis of un-treated and treated hybrid PN models

**Fig 10:**
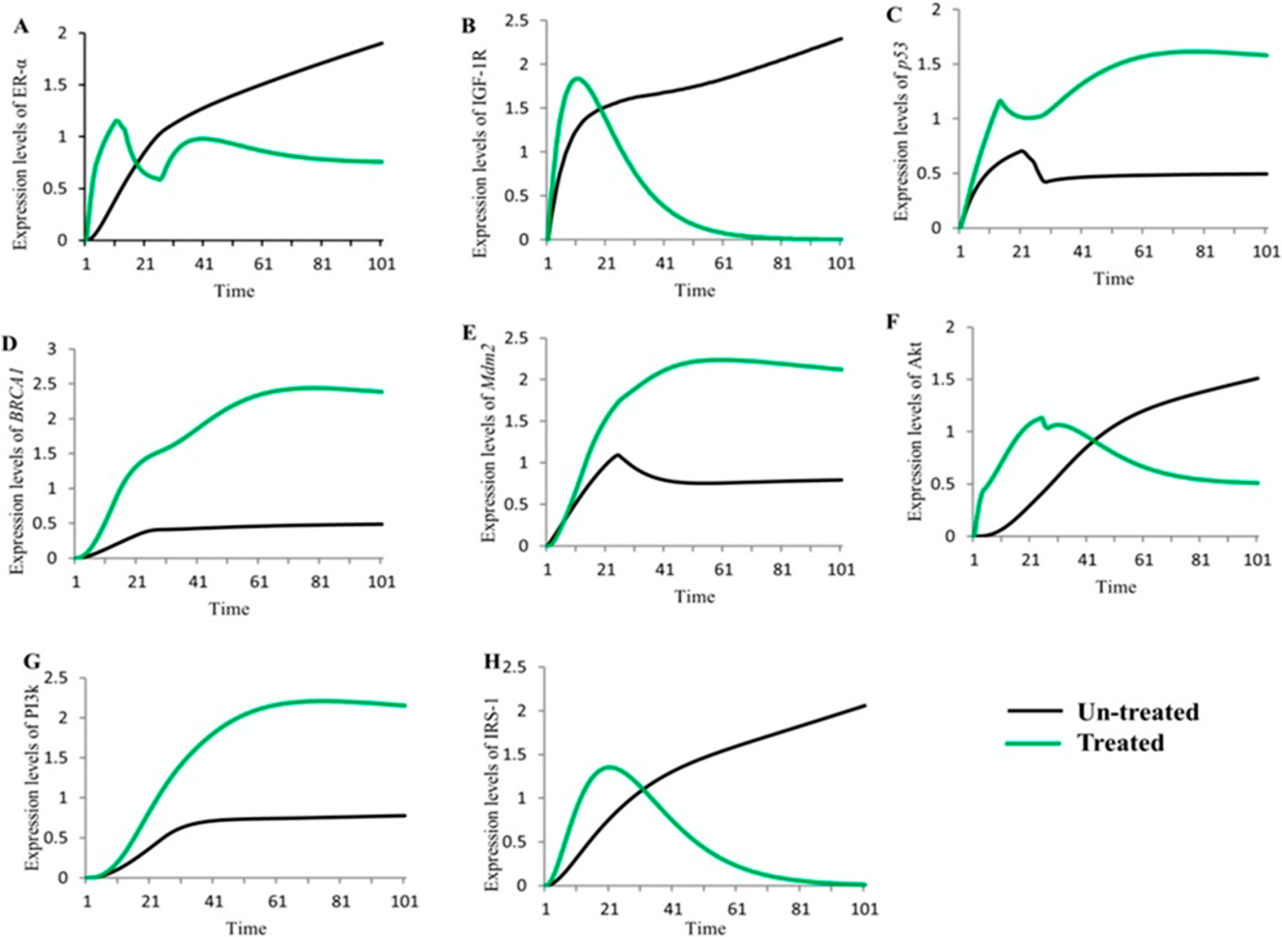
Comparison of simulated graphs of both un-treated and treated IGF-1R associated HPN models. The simulation graph represents the relative expression level (*x*-axis) of key entities with respect to time (*y*-axis). The black sigmoid curves represent the un-treated behaviours and green curves represent the treated behaviours of entities involved in IGF-1R associated signaling. Fig 10A–10H represents the relative change in dynamical behaviours of key proteins (ER-α, IGF-1R, p53, BRCA1, Mdm2, Akt, PI3k and IRS-1) before and after treated with anti-estrogen to be occurred.

The comparison analysis was performed to show the simulation results of IGF-1R associated signaling genes/proteins (ER-α, IGF-1R, p53, BRCA1, Mdm2, Akt, PI3k and IRS-1) with both un-treated (represented by black sigmoid curves) and treated with fulvestrant (represented by green sigmoid curves) hybrid PN models with respect to time (Fig 10). Previously, it has been observed that IGF-1R and ER-α serve as an important inhibitory targets to control breast cancer pathogenesis [20, 98, 99]. Our results are in line with these previous experimental findings [6, 19, 23, 73, 85, 86, 100]. The differences in expression levels of proteins shown in Fig 10A–10F based on our interpretation of the results performed by hybrid PN modeling. In the un-treated model, proteins (ER-α, IGF-1R, Akt and IRS1) were shown to be over-expressed which leads towards breast cancer metastasis (Fig 10A, 10B, 10F and 10H). While the treated model, were shown the controlled expression of proteins which results in the regulation of TSGs (Fig 10C, 10D and 10E). These TSG are helped in maintaining homeostasis.

### Fulvestrant reduces the %age viability of breast cancer cells

**Fig 11:**
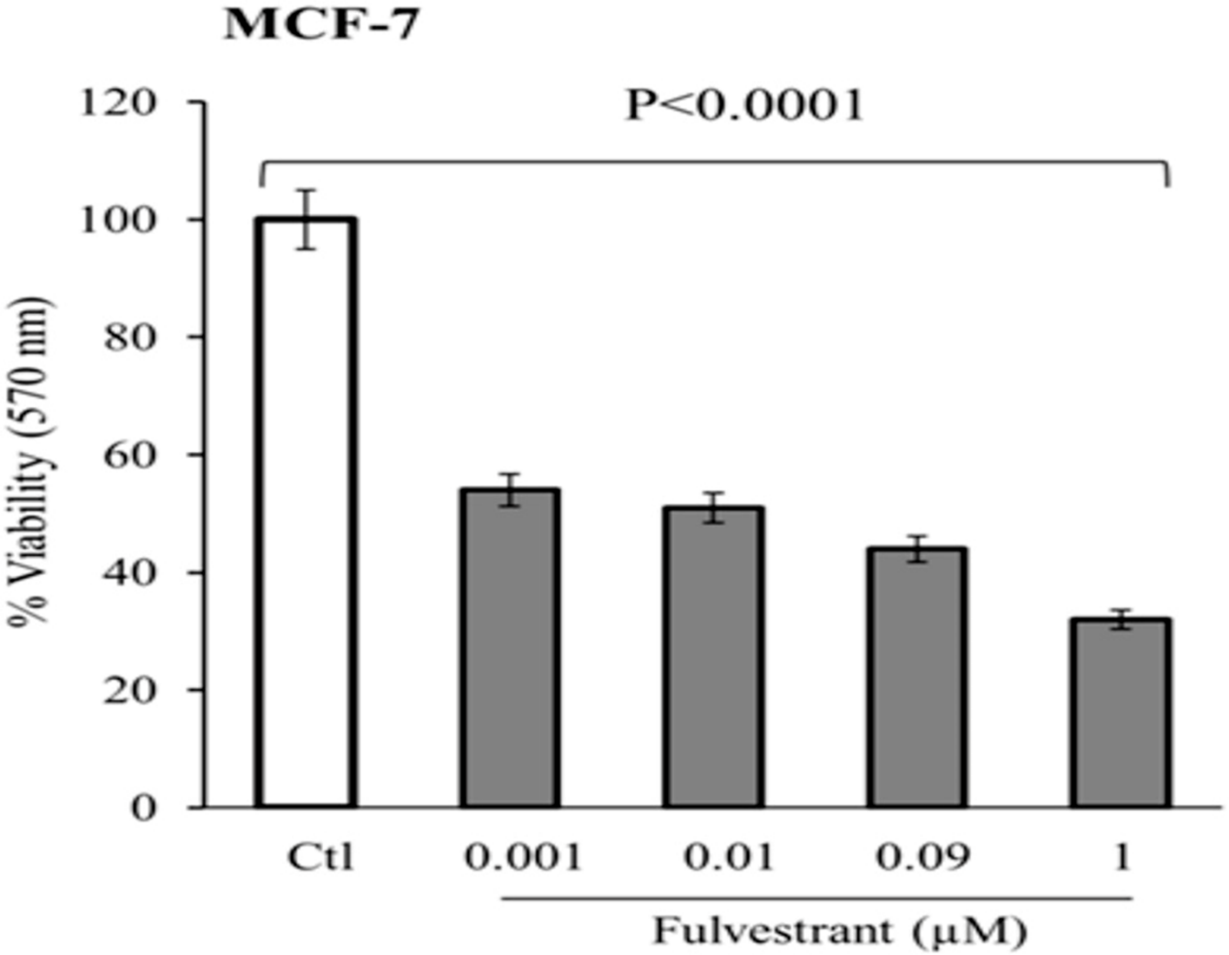
Dose dependent effect of fulvestrant on cell viability. MTT assay of MCF-7 cells treated with various concentration of fulvestrant. Statistically significant reduced the % viability in MCF-7 cells (One-way ANOVA; ****p < 0.0001).

In the present study, cell viability was determined in MCF-7 (ATCC, Manassas, VA) cell line by MTT assay, treated with various concentrations (0.001–1μM) of fulvestrant (Faslodex, AstraZeneca, USA). Previous studies have described the mechanism of interaction of fulvestrant in MCF-7 cells to stop cell cycle proliferation [101–103] and reduced expression levels of estrogen regulated genes/proteins involved in breast cancer progression [79]. IC_50_ value of fulvestrant was calculated as 0.02 μM and its regression analysis showed that large R-square (R^2^) value of 0.88 for linear model was established under good fit. The p value was considered statistically significant (p < 0.0001) (Fig 11). It shows 50% significant reduction in the viability of MCF-7 cells in a dose dependent manner as compared to control. In contrast, fulvestrant at same concentration was only able to reduce cell viability to 110, 101, 95 and 92% respectively, in human corneal epithelial cells (HCEC) (*data not shown*). These results provide that fulvestrant has selective cytotoxic effect in MCF-7 cells without damaging normal cells.

### Treatment with fulvestrant decreased mRNA expression of target genes IGF-1R, PDZK1 and ER-α in MCF-7 cells

**Fig 12:**
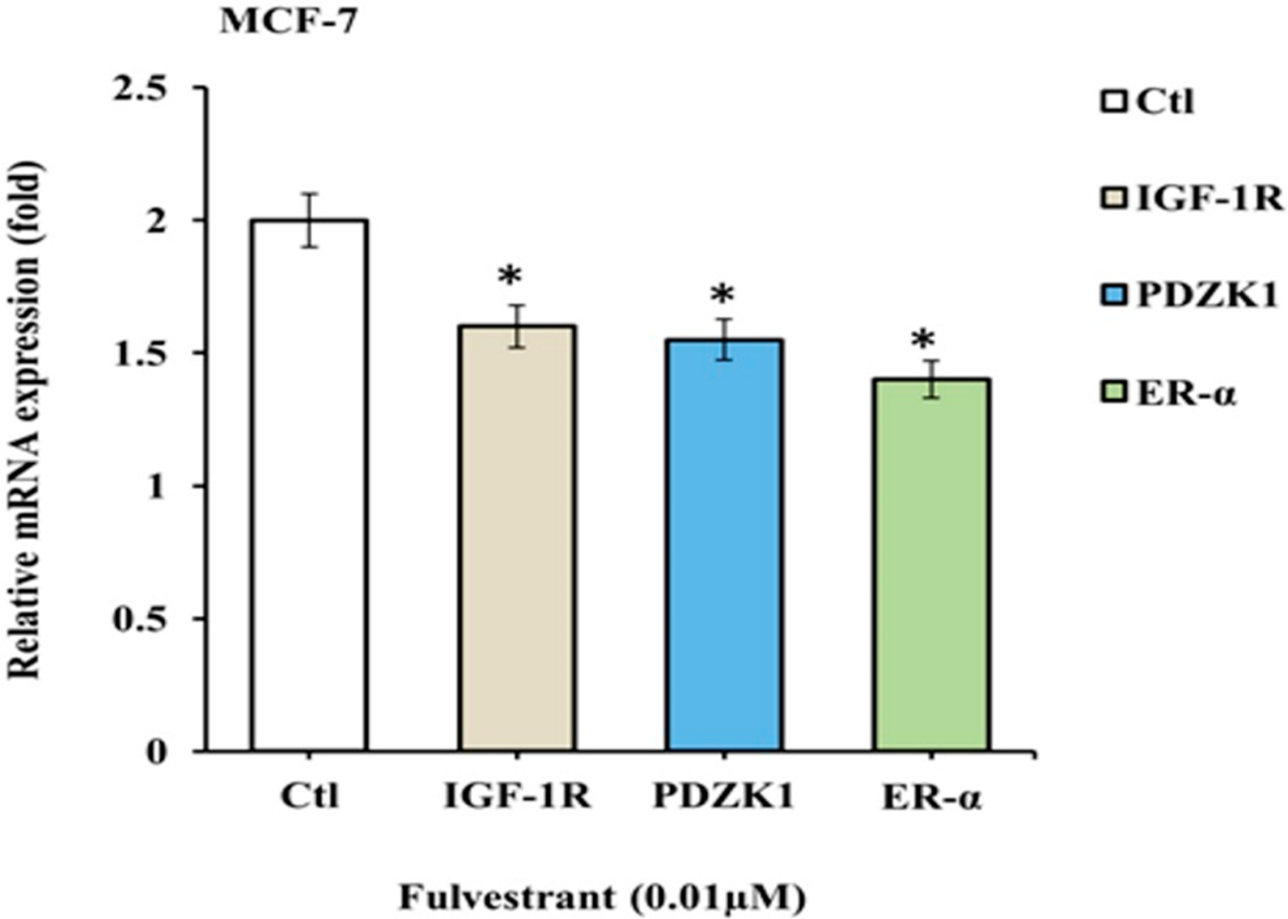
IGF-1R, PDZK1 and ER-α mRNA levels in MCF-7 cell line treated with fulvestrant: MCF-7 cells were treated with 0.01μM fulvestrant for 48hrs prior to RNA preparation. β-actin was assessed as internal control. Data was analyzed by Graph Pad Prism and asterisks shows the statistical significance of genes IGF-1R, PDZK1 and ER-α (p<0.05) in treated cultured medium in MCF-7 cells.

MCF-7 cells were treated with drug fulvestrant and mRNA levels of IGF-1R, PDZK1 and ER-α gene was quantified by quantitative real-time (q-RT-PCR). A significant decrease (1.6, 1.55 and 1.4) was observed in the expressions of IGF-1R, PDZK1 and ER-α genes treated with fulvestrant (shown in Fig 12). The role of ER-α in metastasis of breast cancer has been investigated and considered as important inhibitory target to control cell proliferation [20]. The metastatic response to PDZK1 and ER-α was reduced significantly in MCF-7 cells in which over-expression of IGF-1R had been decreased (Fig 12). Subsequent to the reduction in IGF-1R expression levels resulted in reduce expressions of ER-α and PDZK1, associated with decreased risk of developing breast cancer metastasis.

### Treatment with fulvestrant decreased protein expression of IRS-1, IGF-1R, PDZK1 and ER-α in MCF-7 cells

**Fig 13:**
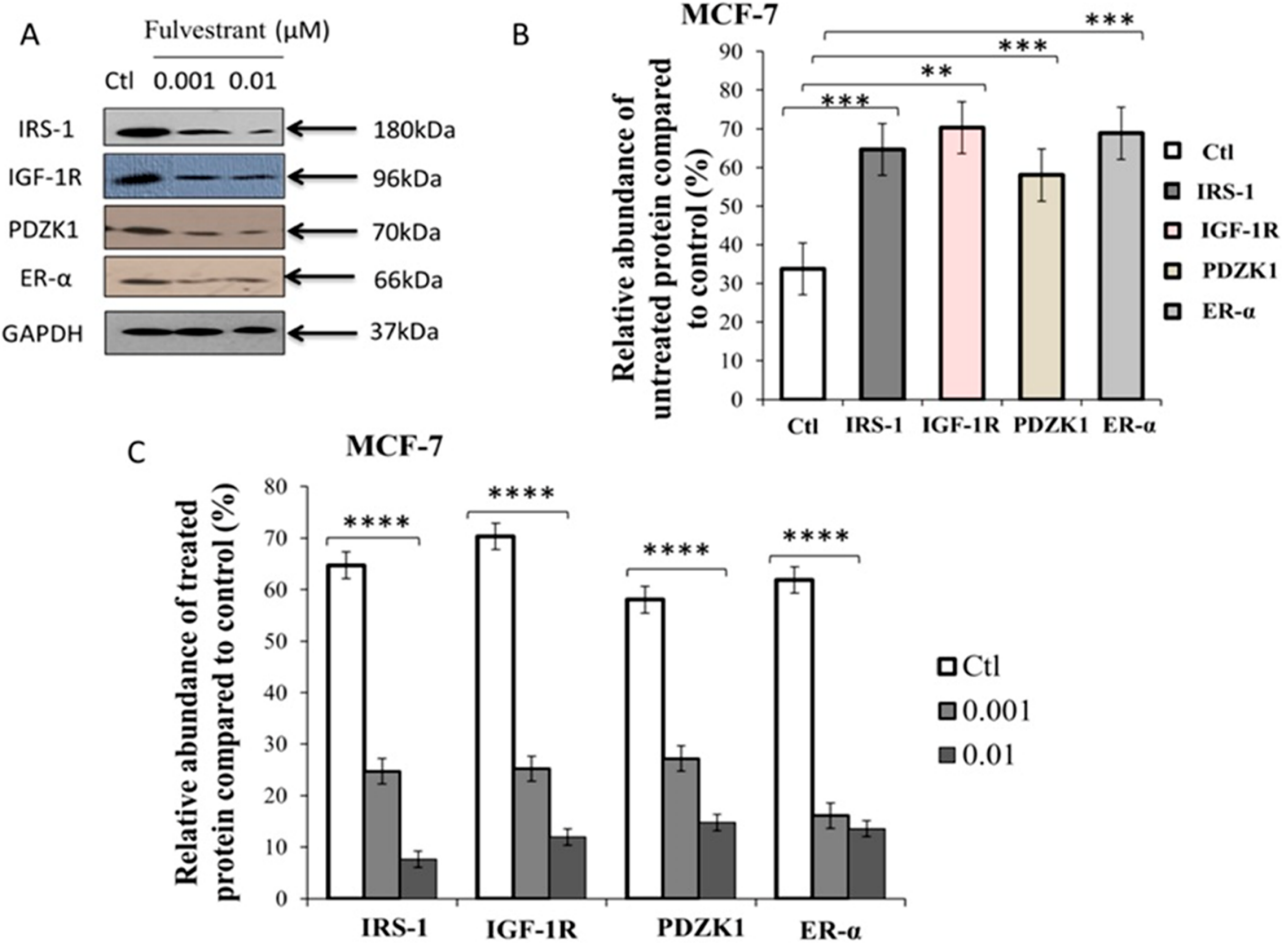
Western immunoblots shows dose dependent response of fulvestrant on relative abundance of proteins (IRS-1, IGF-1R, PDZK1 and ER-α) in MCF-7 cell lysates. (A) Lane 1 shows the control. Lane 2, 3 represents the protein expression profile of breast cancer cell line and following treatment with fulvestrant (0.001–0.01) for 48hrs. GAPDH was assessed as a loading control. Cells were incubated with antibodies and fixed against IRS-1, IGF-1R, PDZK1 and ER-α. Protein bands were measured and normalized to GAPDH or total individual protein expression. (B) Relative expressions of proteins compared with loading control. Asterisks shows the statistical significance of proteins cultured in serum containing medium in MCF-7 cells (unpaired *t* test; IRS-1 ***p < 0.0002, IGF-1R **p < 0.0011, PDZK1 ***p < 0.0004 and ER-α ***p < 0.0008). (C) Relative abundance of dose dependent proteins was expressed as %age of maximum value measured as means ± SEM from three replicate for each blot experiment. Asterisks indicate the statistical significance of proteins cultured in serum containing treated medium in MCF-7 cells (One-way ANOVA; *p < 0.01).

Dose dependent treatment of ER+ MCF-7 cells with fulvestrant (0.001–0.01μM) for 48hrs shows the reduced expression levels of proteins such as IRS-1, IGF-1R, PDZK1 and ER-α (Fig 13A). Previously, fulvestrant has been reported to down-regulate the expression of PI3k in combination with the inhibitors such as *BYL719*, *GDC-0941*, *GDC-0980* and *BKM120* [104]. Fig 13B represents the relative expression levels of un-treated proteins which were significantly higher: IRS-1 (p = 0.0002), IGF-1R (p = 0.001), PDZK1 (p = 0.0004) and ER-α (p = 0.0008) in breast cancer cells. This result is consistent with hybrid PN modeling (see section analysis of hybrid PN modeling of IGF-1R associated diseased regulatory network) showed in this study which observed the over-expression of IRS-1, IGF-1R and ER-α by down-regulating the expression of TSGs. PDZK1 is one of the important therapeutic target in breast cancer treatment that connects strong relationship with ER-α stimulated through direct interaction with IGF-1R [26]. Hence, it is important to note that the up-regulated expressions of all these proteins based on both *in-silico* and *in-vitro* analysis, serves as an important inhibitory targets for metastatic breast cancer treatment. A dose dependent response of fulvestrant was measured statistically significant to p<0.0001 on relative abundance of proteins compared with loading control (Fig 13C). It shows the up-regulated expressions of all proteins are relatively controlled by low amount of fulvestrant tend to reduce the cellular proliferation and metastasis. Our results suggest that fulvestrant is an effective drug that inhibits the pathogenic/carcinogenic effects of estrogen dependent IGF-1R, IRS-1, ER-α and PDZK1 signaling pathways to control breast cancer progression.

### Comparison of expression levels of both *In-silico* and *In-vitro* analysis of un-treated and treated behaviours of proteins in breast cancer signaling

The comparison study of both *in-silico* (given in Figs 7, 9 and 10) and *in-vitro* analysis (given in Fig 13) of un-treated and treated behaviours of proteins IGF-1R, IRS-1 and ER-α showed a strong correlation in our results as presented in Table 3. The similar expression levels of protein IRS-1 was observed through *in-silico* simulations and *in-vitro* western analysis of both un-treated and treated experiments. In breast cancer cells, transcriptional activity of IRS-1 can be increased by steroidal hormones such as estrogen and progesterone respectively in response to IGF-1 [105, 106]. Previously studies have showed that IRS-1 was highly expressed in MCF-7 cells and sensitized to specific chemotherapeutic agents [106–108]. We found that reducing IRS-1 levels in breast cancer cells resulted in a reduced expression of ER-α. In un-treated cells, the over-expression of IGF-1R (represented by +++) considered as most active protein in *in-silico* modeling as compared to western analysis (represented by ++) was observed. In contrast, ER-α shows the moderately active protein (represented by ++) compared to wet-lab experiments which means that there is a crosstalk between IGF-1R and ER-α, associated with an increased risk of breast cancer metastasis. These results confirm the previous studies demonstrating cross-regulation between IGF-1R and IRS-1 [6, 19], IGF-1R and ER-α [6], IRS-1 and ER-α [107, 109]. High expression of IGF-1R due to phosphorylated activity of IRS-1 can enhanced activity of ER-α which leads the tumor towards metastasis. Furthermore, the association of IGF-1R with ER-α in response to IRS-1 within the nucleus and modulates its transcriptional activity at estrogen responsive genes [20, 107, 109]. Our *in-silico* and *in-vitro* studies have shown relative decrease in expression levels of IGF-1R, IRS-1 and ER-α (represented by—) by the biological activity of targeted drug. Fulvestrant should be used to inhibit multiple targets (IGF-1R, IRS-1, PDZK1 and ER-α) involved in breast cancer progression.

**Table 3:** Comparison of expression levels of both *In-silico* and *In-vitro* analysis related to IGF-1R associated un-treated and treated signaling proteins. The triple positive sign (+++) indicates the most active, double positive (++) indicates the moderately active and triple negative (---) indicates the down-regulate expression levels of entities in breast cancer cells.

Proteins	Un-treated		Treated	
	*In-silico* simulations	*In-vitro* western analysis	*In-silico* simulations	*In-vitro* western analysis
**IGF-1R**	+++	++	---	---
**IRS-1**	+++	+++	---	---
**ER-α**	++	+++	---	---

## Discussion

Worldwide, the most frequently diagnosed cancer among females is breast cancer [110]. It is a heterogeneous disease caused by a combination of environmental and genetic factors [111]. In ER+ breast cancer treatment, tamoxifen is used as a targeted adjuvant therapy for all the stages [112]. It is a selective ER modulator (SERM) that inhibits estrogen from binding the ligand but development of resistance towards tamoxifen in any stages of breast cancer is very common [112]. The mechanism of resistance to SERMs and other steroidal and non-steroidal classes of drugs such as aromatase inhibitors are increased by disruption in the growth factors (IGFs, EGF) signaling pathways [113–115]. Previously, in the clinical trials, various strategies (monoclonal antibodies against IGF-1R, monoclonal antibodies against IGF-1R ligands (IGF-1 and IGF-2), and IGF-1R tyrosine kinase inhibitors) have been used to control the expression levels of IGF-1R which gets over expressed in about 50% of the cases of breast cancer [15]. The aim of current study was to adopt a more comprehensive strategy by identifying new or/and known compounds (the hits mentioned in the paper) which have the potential to complete blockade IGF-1R and its signaling network (ligand and downstream signaling molecules).

In the present study, we selected predictive features to build a proficient pharmacophore model (Fig 3). It was used to screen the WDB database to short list potent inhibitory drugs (DB00294, DB00304, DB00947, DB07757, DB07150, DB07230, DB06973 and DB07712) (Fig 5) against IGF-1R. Etonogestrol also known as implanon has been revealed to induce mild insulin resistance (IR) but effects are not clinically significant for healthy females [116]. One of the oral contraceptive methods is sub-dermal contraceptive implant (SCI) used in combination with etonogestrol, the synthetically active metabolite of desogestrol [117]. However, in contrast to etonogestrol and desogestrol, fulvestrant is selective estrogen receptor down regulator (SERD) that prevents dimerization of ER and proves to be effective in breast cancer treatment [79, 118]. This effect leads to abridged crosstalk signaling between ER and ER-independent growth factor signaling pathway, IGF, thus delaying resistance to endocrine treatment [118]. The results of MTT assay showed that fulvestrant significantly decreased cell viability (%) of MCF-7 cells in a dose dependent manner (Fig 11). It was observed that fulvestrant exhibits significantly selective cytotoxicity in breast cancer as compared to normal HCECs at the same concentration. Previous studies have reported that ER antagonist fulvestrant has the ability to enhance immune and chemotherapeutic-mediated cytotoxicity in lung carcinoma cells [119].

The intense collaboration of *in-silico* methods in drug repurposing (through ligand based pharmacophore modeling and VS) and hybrid PN modeling [120] was performed to find the significance of fulvestrant on activity of proteins involved in IGF-1R signaling. The powerful synergy of PN model (Fig 6) was constructed based on various studies performed in wet lab using techniques such as: reverse phase protein analysis, DNA sequencing and copy number, immunohistochemistry, micro RNA, polymorphisms and western analysis [6, 21, 62–66, 83, 121]. Studies showed that increased expression of estrogen by ligand binding interaction (IGFs with IGF-1R) can regulate the downstream signaling mediators such as IRS-1, Akt and PI3k in breast cancer cells. Our simulation results suggested that the mutated behaviours of *p53*, *BRCA1* and *Mdm2* (represented by dash, round dot and square dot curves) were down-regulated (Fig 7). The significant up-regulated expression of IGF-1R is increased by the suppression of TSGs which lead tumor towards metastasis. It was concluded that high levels of IGF-1R, IRS-1 and ER-α can be controlled by inhibitor, fulvestrant.

The therapeutic role of fulvestrant has demonstrated to be effective in the treatment of locally advanced ER or/and PR positive and HER2 negative breast cancer patients [79]. It gives a more prominent control over endocrine treatment consistence, decreasing oral ingestion and pharmacokinetic interactions with sustenance or other medications, which are vital perspectives to be considered in patients with breast cancer [79]. Due to preferable understanding of biological signaling pathways engrossed in the tumor growth and development, a few trials are assessing the role of new organic medications to blocking regulatory network. Some of the new drugs such as everolimus (mTOR inhibitor) [122], BYL719, GDC-0941, GDC-0980, and BKM120 (PI3k inhibitors) are being evaluated in combination with fulvestrant for the treatment of breast cancer metastasis [79]. Fig 8 describes a treatment algorithms of IGF-1R associated signaling network involved in breast cancer metastasis. The simulation results showed the effective behaviour of inhibitor against IGF-1R, IRS-1 and ER-α which leads the system towards homeostasis. The increased expression of *PTEN* controlling the autophosphorylation and over-expression of hormonal receptors (Fig 9) were observed by hybrid PN modeling. The differences between expression level of both un-treated and treated behaviours of IGF-1R associated entities were clearly seen in comparison study to analyse the inhibitory effect of fulvestrant (Fig 10). The levels of IGF-1R, IRS-1, ER-α and Akt are relatively decreased (represented by green sigmoid curves) by the up-regulated expressions of TSGs as compared to un-treated dynamical behaviours (represented by black sigmoid curves) with respect to time. It is based on our interpretation obtained in this study through *in-silico* modeling and simulations that fulvestrant act as an effective inhibitor against multiple breast cancer inhibitory targets to control the effect of metastasis.

**Fig 14:**
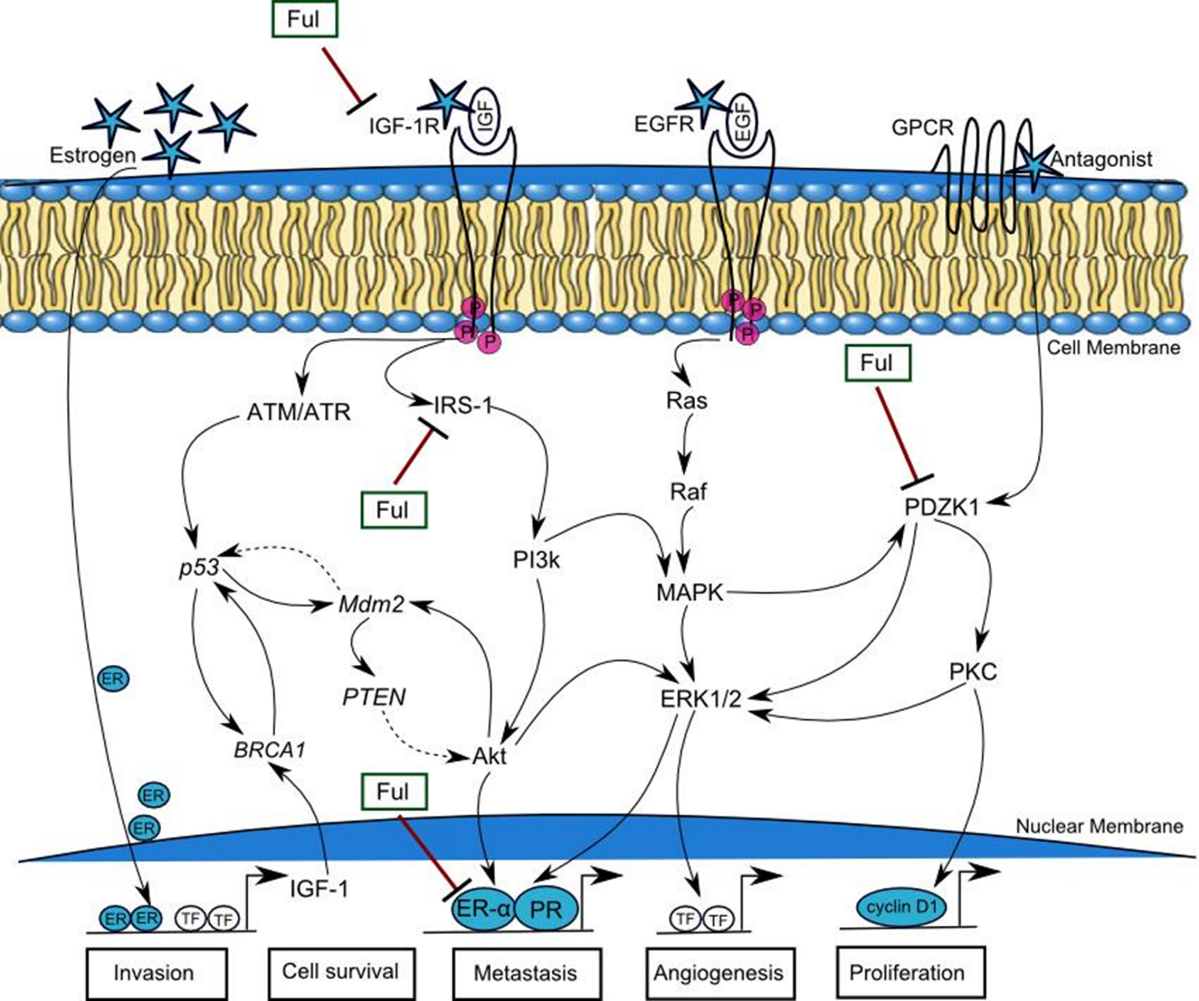
Pathways by which targeting growth factor and estrogen receptor to overcome endocrine therapy resistance in breast cancer. A mechanism begins by the cell signals (represented by stars) transduction through trans-membrane receptors (IGF-1R, EGFR and estrogen) and antagonist from extracellular matrix to nucleus. Estrogen up-regulates the transcription factors (TF) which regulates the activity of growth factor IGF-1. Binding of ligands IGF and EGF with targeted receptors (IGF-1R and EGFR) involve in phosphorylation and trans-activation of several downstream mediator proteins lead the system towards metastasis. G-protein coupled receptor (GPCR) transmits the signal by the binding of isomers (Gα) which enhance the transcription of cyclin D1 by over-expressing PDZK1. The up-regulated expression of growth factors could lead to the activation of estrogen and progesterone receptors (ER, PR). Fulvestrant (Ful) inhibits the phospho-IRS-1, IGF-1R, ER-α and PDZK1, involved in breast cancer invasion, cell survival, metastasis, angiogenesis and cell proliferation.

After ensemble PN model, which is constructed through Kyoto Encyclopedia of Genes and Genomes (KEGG) [123] or literature databases of interactions among genes and proteins [6, 20, 21, 62–66, 83, 113, 121], we selected most effective compound fulvestrant for subsequent wet experiments. The IC_50_ value of fulvestrant was 0.02μM as determined by MTT assay therefore we selected this compound for further investigations. Fulvestrant is currently used in long term estrogen deprivation (LTED) therapy of ER+ breast cancer cells. It was recently identified as a potential anti-cancer which inhibits PI3k-Akt, ERK/MAPK and Janus kinase (JAK)-signal transducer and activator of transcription (STAT) pathways involved in cell survival, proliferation, invasion and drug resistance [103]. Based upon our data, we constructed a resultant mechanism of IGF-1R associated breast cancer cellular pathway by which targeted growth factors to overcome endocrine therapy resistance (Fig 14). The q-RT-PCR and western blot analysis illustrated in Figs 12 and 13, acquire a more aggressive biological phenotype and how fulvestrant interplay in this signaling pathway. In summary, breast cancer cells exhibits activation of multiple growth promoting factors: IGF-1R, IRS-1, PDZK1 and ER-α. They also show an enhanced response to IGF, EGF, ER and antagonist, which in turn activates PI3k-Akt, ATM/ATR, MAPK/ERK1/2 and G-protein coupled receptor (GPCR) signaling pathways, which cross-talk with elevated estrogens. Treatment of cells with fulvestrant significantly inhibits ER-α dependent IGF-1R, phospho-IRS-1 and PDZK1 pathways regulating cancer cell invasion, survival, metastasis, angiogenesis and proliferation. The signaling alterations of MCF-7 cells in turn feed into multiple TSGs including BRCA1, p53, Mdm2 and PTEN which are activated as a result of activated signaling.

There is a strong correlation of PDZK1 with multiple signaling pathways including estrogen dependent IGF-1R and chemokine (C-X-C motif) receptor 4 (CXCR4) signaling which can be targeted therapeutically to treat breast cancer [24, 26, 124]. It has been reported that the over-expression of PDZK1 was associated with resistance to paclitaxel-5-fluorouracil-etoposide at low concentration [24]. In this study, we reported for the first time that fulvestrant exhibited significant inhibitory activity against PDZK1. More importantly, our results demonstrate a novel function for PDZK1 and find a molecular crosstalk between biological regulatory growth factors that are involved in breast cancer signaling. Although, we tested fulvestrant *in-silico* and *in-vitro* in MCF-7 cells, same biological results were observed to analyse the deregulated expression of growth promoting factors (Table 3). It shows the inhibitory effect of fulvestrant on IGF-1R, IRS-1, ER-α and PDZK1 at low concentration. We believe, that fulvestrant may exhibit as an effective and safe anti-cancer drug against multiple targets specially IGF-1R but further experimental validation is needed using inhibition assay against this receptor’s activity and its downstream signaling molecules which should be elucidated in the future studies.

## Supporting information

S1 Table Pharmacophore model was validated by the significance of statistical parameters with respect to molecular sequence and root mean square deviation (RMSD). It shows the RMSD of 21 set of active compounds with respect to IC_50_ values were generated to build the pharmacophore model. The low RMSD values have ability to predict the activity of the conformational dataset compounds.
(DOCX)
