# Supplementary material for: Pharmacophore modeling for identification of anti-IGF-1R drugs and in-vitro validation of fulvestrant as a potential inhibitor
Source: PLoS One. 2018 May 22;13(5):e0196312. doi: 10.1371/journal.pone.0196312 (PMC5963753; doi:10.1371/journal.pone.0196312)
Supplement: S1 Table — It shows the RMSD of 21 set of active compounds with respect to IC50 values were generated to build the pharmacophore model. The low RMSD values have ability to predict the activity of the conformational dataset compounds. (DOCX) [file pone.0196312.s001.docx]

Supporting Information 1.

S1 Table. Pharmacophore model was validated by the significance of statistical parameters with respect to molecular sequence and root mean square deviation (RMSD). It shows the RMSD of 21 set of active compounds with respect to IC_50_ values were generated to build the pharmacophore model. The low RMSD values have ability to predict the activity of the conformational dataset compounds.

| **S.No.** | **IC_50_ (µM)** | **Molecular sequence** | **RMSD** |
| --- | --- | --- | --- |
| 1 | 0.04 | 1 | 0.0102 |
| 2 | 2.77 | 13 | 0.0324 |
| 3 | 20 | 14 | 0.0351 |
| 4 | 31 | 18 | 0.0463 |
| 5 | 6 | 20 | 0.0717 |
| 6 | 0.5 | 4 | 0.0778 |
| 7 | 10 | 7 | 0.0936 |
| 8 | 22 | 16 | 0.1282 |
| 9 | 60 | 21 | 0.1554 |
| 10 | 1.8 | 6 | 0.1836 |
| 11 | 14 | 11 | 0.2059 |
| 12 | 13.5 | 10 | 0.3916 |
| 13 | 12 | 9 | 0.4450 |
| 14 | 1 | 5 | 0.4537 |
| 15 | 0.25 | 2 | 0.4652 |
| 16 | 40 | 19 | 0.4856 |
| 17 | 20 | 15 | 0.7056 |
| 18 | 0.3 | 3 | 0.7642 |
| 19 | 25.6 | 17 | 0.8980 |
| 20 | 10.4 | 8 | 0.8983 |
| 21 | 16.2 | 12 | 0.8983 |
